# Effect of Pterostilbene, a Natural Analog of Resveratrol, on the Activity of some Antiepileptic Drugs in the Acute Seizure Tests in Mice

**DOI:** 10.1007/s12640-019-00021-1

**Published:** 2019-03-15

**Authors:** Dorota Nieoczym, Katarzyna Socała, Piotr Jedziniak, Elżbieta Wyska, Piotr Wlaź

**Affiliations:** 1 Department of Animal Physiology, Institute of Biology and Biochemistry, Faculty of Biology and Biotechnology, Maria Curie-Skłodowska University, Akademicka 19, 20-033 Lublin, Poland; 2 Department of Pharmacology and Toxicology, National Veterinary Research Institute, Puławy, Poland; 3 Department of Pharmacokinetics and Physical Pharmacy, Collegium Medicum, Jagiellonian University, Kraków, Poland

**Keywords:** Pterostilbene, Antiepileptic drugs, Pharmacological interactions, Seizure models, Mice

## Abstract

Pterostilbene (PTE), a natural analog of resveratrol, is available both as a diet ingredient and a dietary supplement. The present study was undertaken to assess the effect of PTE on the activity of antiepileptic drugs in the acute seizure tests in mice, i.e., the intravenous pentetrazole (*iv* PTZ) seizure threshold, maximal electroshock (MES), and 6 Hz-induced psychomotor seizure tests. Our study revealed that PTE enhanced the anticonvulsant effect of clonazepam but did not change the activity of tiagabine in the *iv* PTZ test. In the MES test, PTE increased the effect of carbamazepine but did not affect the protective properties of topiramate, while in the 6-Hz test, we noted a significant enhancement of the activity of oxcarbazepine, but there were no changes in the activity of valproate. Interactions of PTE with carbamazepine and oxcarbazepine were pharmacokinetic, which was determined by the increase of concentration of these antiepileptic drugs both in the serum and brain. In contrast, interactions between PTE and clonazepam were pharmacodynamic since there were no changes in the concentration of clonazepam. Combined treatment with carbamazepine and PTE significantly attenuated muscular strength (estimated in the grip strength test) but did not change motor coordination (assessed in the chimney test) in mice. Other studied antiepileptic drugs and their combinations with PTE did not change these parameters. Further studies are required to evaluate the influence of PTE on the activity of anticonvulsant drugs to estimate the safety of using PTE by patients with epilepsy.

## Introduction

Pterostilbene (PTE, 3,5-dimethoxy-4′-hydroxystilbene) is a naturally occurring polyphenol in the stilbene group which was first isolated from red sandalwood and subsequently detected in grapes, blueberries, and heartwood. It was also identified in some plants used in folk medicine for the treatment of diabetes and cardiovascular diseases (Kosuru et al. 2016). PTE is a natural analog of resveratrol (RES), the most popular and known stilbenoid, which was detected in red wine and recognized as one of the compounds responsible for “French paradox” (Poulose et al. 2015). Compared with RES, PTE has greater lipophilicity and higher bioavailability after oral administration, which makes it more clinically useful (Kapetanovic et al. 2011). Both of these compounds produce numerous health beneficial effects, e.g., antioxidative, anti-inflammatory, anticancer, and anti-obesity properties (Kosuru et al. 2016).

A wide range of biological and pharmacological effects of PTE has attracted attention from the scientific and medical community and suggests that this compound might have some beneficial effects in cardiovascular diseases, cognitive dysfunctions, and aging (Akinwumi et al. 2018). Diet supplemented with PTE reversed the decline of cognitive performance in aged rats (Joseph et al. 2008). Moreover, PTE abolished streptozotocin-induced memory impairment in rats (Naik et al. 2017) and lipopolysaccharide-induced learning and memory deficits in mice (Hou et al. 2014). Its anxiolytic activity was observed in the elevated plus maze test in mice (Al Rahim et al. 2013). Our recent study demonstrated an anticonvulsant activity of PTE both in the zebrafish pentetrazole (PTZ) assay and in three acute seizure tests in mice, i.e., in the intravenous (*iv*) PTZ seizure threshold, maximal electroshock seizure threshold (MEST) and 6 Hz seizure threshold tests (Nieoczym et al. 2019). A human clinical trial revealed that therapy with PTE decreases both systolic and diastolic blood pressure as well as increases low-density lipoprotein (LDL) concentration (Riche et al. 2014). Clinical study did not show any negative drug effects on the hepatic, renal, and glucose markers (Riche et al. 2013).

PTE is a constituent of the human diet and is currently accessible as a diet supplement recommended to patients with heart diseases, high blood pressure, and diabetes. Moreover, PTE might improve memory, reduce stress, and anxiety as well as support weight loss (Li et al. 2018). Market accessibility of PTE makes a possibility for taking it by patients with epilepsy, also simultaneously with antiepileptic drugs. To study the interaction between PTE and antiepileptic drugs, we were also prompted by the results of our previous study that demonstrated the anticonvulsant properties of PTE. Combined treatment with antiepileptic drugs and PTE creates an opportunity for some interactions between these compounds, which might have beneficial or negative results. Investigation of the interactions between antiepileptic drugs and PTE is necessary to determine the safety of the studied stilbene and to estimate potential benefits or wastes of using PTE as an added therapy in patients with epilepsy.

The aim of the present study was to investigate the influence of PTE on the anticonvulsant activity of some antiepileptic drugs in three acute seizure tests in mice, i.e., clonazepam (CZP) and tiagabine (TGB) in the *iv* PTZ test, carbamazepine (CBZ), and topiramate (TPM) in the maximal electroshock (MES) test, valproate (VPA) and oxcarbazepine (OXC) in the 6 Hz-induced psychomotor seizure test. For each test we selected two drugs—one medicine from the first generation (i.e., CZP, CBZ and VPA) and one second-generation drug (i.e., TGB, TPM, and OXC), which are characterized by a high effectiveness in inhibiting convulsions induced in a given model (Barton et al. 2001; Löscher and Schmidt 2011). Additionally, total brain and free serum concentrations of CZP, CBZ, and OXC were determined to define the kind of their pharmacological interaction with PTE. Influence of the studied antiepileptic drugs and their combinations with PTE on the motor coordination and muscular strength in mice were estimated in the chimney and grip strength tests, respectively.

## Material and Methods

### Animals

Male Swiss mice weighing 23–28 g were obtained from a licensed breeder (Laboratory Animals Breeding, Słaboszów, Poland) and used in the study after at least 1 week of acclimatization. The animals were housed in the polycarbonate cages under strictly controlled conditions (ambient temperature 21–24 °C, relative humidity 45–65%, a 12/12 light/dark cycle with the light on at 6:00 a.m.) with unlimited access to chow pellets and tap water. All experiments were performed at the same time of day (between 8:00 a.m. and 3:00 p.m.) to minimize circadian influences. Control and drug experiments were always done on the same day to avoid day-to-day variations in convulsive susceptibility. Each mouse was used only once. All procedures were conducted in accordance with the European Union Directive of 22 September 2010 (2010/63/EU) and Polish legislation acts concerning animal experimentations. The experimental procedures and protocols were approved by the Local Ethics Committee in Lublin (License No. 18/2018).

### Drugs

The following drugs were used: PTE (Toronto Research Chemicals INC, Toronto, ON, Canada), CZP (Clonazepamum, Polfa, Warszawa, Poland), TGB (Gabitril, Sanofi Winthrop, Gentilly, France), CBZ (kindly donated by Polpharma S.A., Starogard Gdański, Poland), TPM (Topiran, Ranbaxy, Warszawa, Poland), VPA (as sodium salt; Sigma-Aldrich Co., St. Louis, MO, USA), and OXC (Trileptal, Novartis Pharma GmbH, Nümberg, Germany). VPA was dissolved in saline, while the remaining drugs were suspended in a 5% solution of Tween 80 (POCH, Gliwice, Poland) in normal saline. All the used solutions/suspensions were administered intraperitoneally (*ip*) at a constant volume of 10 ml per kg body weight. VPA and TGB were injected 15 min before the respective experimental procedure, PTE, CBZ, OXC, and CZP 30 min, while TPM 60 min before the tests. The pretreatment times for PTE and the studied antiepileptic drugs were taken from the literature and confirmed in our previous studies (Nieoczym et al. 2010, 2013, 2019).

### The Timed *iv* PTZ Test

Mice were placed in the cylindrical restrainer, and the needle (27 G, 3/4 in., Sterican®, B. Braun Melsungen AG, Melsungen, Germany) was inserted into the lateral tail vein. Correct location of the needle was validated based on the appearance of the blood in the drain and, to avoid its displacement, the needle was stuck by a piece of adhesive tape to the tail. The needle was attached by the polyethylene drain (PE20RW, Plastic One Inc., Roanoke, VA, USA) with the syringe which was filled with 1% solution of PTZ (Sigma-Aldrich, St. Louis, MO, USA) in water and put in the infusion pump (model Physio 22, Hugo Sachs Elektronik–Harvard Apparatus GmbH, March-Hugstetten, Germany). The syringe pump infused the PTZ solution into the vein at a constant rate of 0.2 ml/min. Following the injection, mice were taken out from the restrainer and placed individually in the Plexiglas box for observation. During the infusion of PTZ solution into the vein of freely moving animals, three seizure types sequentially appeared, i.e., the first myoclonic twitches, generalized clonus with loss of righting reflex, and forelimb tonic extension. The infusion of PTZ was stopped immediately after the onset of the tonic seizures, which usually were lethal. If an animal survived the test, it was euthanized immediately. Time intervals from the start of infusion to the appearance of the three separate types of seizures were noted, and the threshold doses of PTZ (in mg/kg body weight) for each type of seizures were calculated according to the following formula:

$$ \mathrm{PTZ}\ \left(\mathrm{mg}/\mathrm{kg}\right)=\frac{\mathrm{infusion}\ \mathrm{duration}\ \left(\mathrm{s}\right)\times \mathrm{infusion}\ \mathrm{rate}\ \left(\mathrm{ml}/\mathrm{s}\right)\times \mathrm{PTZ}\ \mathrm{concentration}\ \left(\mathrm{mg}/\mathrm{ml}\right)}{\mathrm{weight}\ \left(\mathrm{kg}\right)} $$

Experimental groups consisted of 9–13 animals. The data obtained in the *iv* PTZ test are presented as the mean threshold dose of PTZ (in mg/kg) ± standard error of the mean (SEM) needed to elicit the respective endpoint. The data were analyzed by a one-way analysis of variance (ANOVA) followed by the Tukey post hoc test. Statistical significance was noted when a *p* value was equal to or less than 0.05.

### The Maximal Electroshock Seizure Test

The MES test was carried out to evaluate the influence of PTE on the anticonvulsant activity of CBZ and TPM. This test allows the estimation of the median effective doses (ED_50_; the dose which protects 50% of the tested animals form the seizures) of the studied antiepileptic drugs. Animals (3–5 groups; 8 animals/group) were treated with increasing doses of the respective antiepileptic drug or its combinations with PTE, and they were subjected to the stimulation. Stimuli (25 mA, 0.2 s, 50 Hz) were generated by a constant current stimulator (Rodent Shocker, Type 221; Hugo Sachs Elektronik, Freiburg, Germany) and delivered by saline-soaked transcorneal copper electrodes. Before the stimulation, 1% solution of tetracaine hydrochloride was applied into animals’ eyes for local anesthesia. During the stimulation, animals were restrained manually, and immediately after the stimulation, they were placed in the Plexiglas box for observation. The endpoint in the test was the maximal electroconvulsion which was defined as an extension of the hindlimbs above a 90° angle to the torso of the animal. The percentage of animals protected from the tonic hindlimb extension was noted, and ED_50_ doses (in mg/kg) of the tested antiepileptic drugs were calculated according to the log-probit method (Litchfield and Wilcoxon 1949).

The respective ED_50_ values were compared with one-way ANOVA followed by the Tukey post hoc test.

### The 6 Hz Psychomotor Seizure Test

Psychomotor (limbic) seizures were induced by transcorneal supramaximal stimulation (6 Hz, 0.2 ms rectangular pulse, 3 s duration) generated by a Grass S48 stimulator coupled with a constant current unit CCU1 (both from Grass Technologies, West Warwick, RI, USA). To evaluate the influence of PTE on the anticonvulsant potential of VPA or OXC, groups of animals (3–5 groups, 8 animals/group) were treated with increasing doses of the respective antiepileptic drug or its combinations with PTE and stimulated with the fixed supramaximal current intensity of 32 mA. Before stimulation, a drop of ocular anesthetic, 1% tetracaine hydrochloride solution, was applied into the animals’ corneas. The electrodes were soaked in saline to ensure good electrical contact. During the stimulation, animals were restrained manually and immediately after the stimulation were placed in a Plexiglas box for observation of presence or absence of the psychomotor seizures. These seizures were characterized by immobilization or stun, often associated with rearing, forelimb clonus, twitching of vibrissae, and elevated tail (Straub-tail), lasting at least 10 s from the stimulation. Lack of the above symptoms or their cessation within 10 s from the stimulation was considered as absence of seizure activity. Percentage of animals protected from convulsive activity was noted to determine ED_50_ doses. As in the MES test, the log-probit method (Litchfield and Wilcoxon 1949) was used to determine ED_50_ doses of the studied drugs.

One-way ANOVA followed by the Tukey post hoc test was used to compare the respective ED_50_ values, and *p* value equal to or less than 0.05 was considered statistically significant.

### The Grip-Strength Test

Effect of the studied antiepileptic drugs and their combinations with PTE on the skeletal muscles strength in mice was determined in the grip strength test (Meyer et al. 1979). The apparatus (BioSeb, Chaville, France) consisted of a steel wire grid (8 × 8 cm) connected to an isometric force transducer. Each mouse was lifted by the tail and allowed to grasp the grid with its forepaws and then it was gently pulled backward until it released the grid. The maximal force in newtons (N) exerted by a mouse before leaving the grid was recorded. The mean of three consecutive measurements for each animal was calculated and normalized to body weight (mN/g).

Experimental groups consisted of 10–12 animals. Results obtained in the grip-strength test were compared using one-way ANOVA with the Tukey post hoc test.

### The Chimney Test

The chimney test (Boissier et al. 1960) was performed to evaluate the influence of the studied antiepileptic drugs and their combinations with PTE on motor coordination in mice. During the test, mice had to climb backwards up the plastic transparent cylinder (inner diameter 3 cm and length 30 cm) with a coarse inner surface, which was positioned vertically. The inability of the mice to escape from the cylinder within 60 s was considered as motor impairment. Results obtained in the test were presented as the percentage of impaired mice in groups of 10–12 animals and were compared with the Fisher exact probability test.

### Determination of Serum and Total Brain Concentration of CZP

The concentration of CZP was determined in serum and brain tissue with modification of a method published by Gu et al. (2009). The 150 μl of the homogenized sample was spiked the 10 μl of internal standard solution (CZP-D4, 1 μg/ml). The analyte was extracted with 150 μl of acetonitrile and methanol solution (1:1, *v*:*v*). The sample was vortex mixed (1 min, Heidolph vortex, Germany) and centrifuged (14,800 rpm, Sigma ultra-centrifuge, Germany). Next, 150 μl of supernatant was transferred to an autosampler vial and diluted with 150 μl of 0.1% formic acid solution. The 15 μl of sample was injected on the LC-MS/MS system—Agilent 1200 chromatograph (Germany) coupled with Sciex API 4000 tandem mass spectrometer (Canada). The chromatographic separation was performed with Synergy RP-Polar column (75 × 2.0mm, 4.0 μm, Phenomenex, USA) coupled with C18 precolumn (SecurityGuard, Phenomenex, USA). The flow rate of the mobile phase “A” (methanol 0.1% HCOOH (5:95, *v*:*v*) and “B” (methanol:0.1% HCOOH (95:5, *v*:*v*) was 0.3 ml/min. The following elution gradient was applied: 0.0–2.5 min (60% A); 2.0–2.5 min (10% A); 2.5–5.0 min (10% A), 5.0–5.5 min (60%A), 5.5–10 min (60% A). The total run time was 10 min. The oven temperature was 40 °C.

The mass spectrometer, equipped with an electrospray (positive mode), has detected the ions with multiple reaction monitoring (MRM) modes. For the CZP, the following transitions were used: 316.1 → 270.2; 316.1 → 241.1; 316.1 → 214 and for CZP-D4: 320.1 → 274.1.

The retention time of the analytes was 6.4 min. The quantitative analysis was performed with the matrix-matched calibration curve (the ratio of the CZP peak areas of the internal standard versus drug concentrations and were linear in the tested concentration range). The limit of quantitation of CZP in serum and brain was 3.3 ng/ml and 0.66 ng/ml respectively. The relative error for accuracy and the coefficient of variation for precision were less than 10%.

### Determination of Serum and Total Brain Concentration of CBZ and OCX

In order to determine CBZ and OXC concentrations, 100 μl of serum sample diluted with 100 μl of water or 200 μl of brain homogenate (1:4, *w*/*v*) in water were spiked with 10 μl of respective internal standards (IS): carbamazepine (10 μg/ml) for oxcarbazepine and butabarbital (50 μg/ml) for oxcarbazepine. To brain homogenate, 0.2 ml NaCl solution (10 g/100 ml) was added, and the samples were vortexed for 15 s. Subsequently, 1 ml aliquots of extraction solvent (dichloromethane for carbamazepine and ethyl acetate for oxcarbazepine) were added to both serum samples and brain homogenates and were vigorously shaken for 20 min (IKA VXR Vibrax, Germany). After centrifugation (Abbott Laboratories, USA)*,* the organic layers of brain samples were transferred into conical tubes and were evaporated to dryness at 37 °C under a stream of nitrogen. The organic layers separated from serum samples containing OXC were washed with 1 ml of 1 M HCl before evaporation. The residues were dissolved in 100 μL of methanol (carbamazepine) or acetonitrile (oxcarbazepine), and 5 μL of these solutions were injected into the HPLC system. The HPLC system (Merck Hitachi, Darmstadt, Germany) consisted of an L-7100 isocratic pump, an L-7200 autosampler, and a UV variable-wavelength K-2600 detector (Knauer, Berlin, Germany). D-7000 HSM software was used for data acquisition and processing. The analysis was performed on a LiChrospher® 100RP-18 column (250 × 4 mm, 5 μm) coupled with a LiChroCART guard column (4.0 × 4.0 mm) (Merck, Germany) with the same packing material. The mobile phase consisting of acetonitrile and water mixed at a ratio of 34:66, *v*/*v* for carbamazepine and 36:64, *v*/*v* for oxcarbazepine was pumped at a flow rate of 1 ml/min. Chromatographic analyses were carried out at room temperature, and the analytical wavelength was 210 nm. The retention times of OXC and IS were approximately 5.8 and 9.1 min, whereas those of CBZ and IS were 11.3 and 6.2 min, respectively. The calibration curves were constructed by plotting the ratio of the peak areas of the studied drugs to their respective IS versus drug concentrations, and they were linear in the tested concentration range. The relative error for accuracy and the coefficient of variation for precision were less than 10%. No interfering peaks were observed in the blank serum or brain homogenate, and PTE did not interfere with the assay.

## Results

### Influence of PTE on the Anticonvulsant Activity of CZP and TGB in the *iv* PTZ Test

**Fig. 1:**
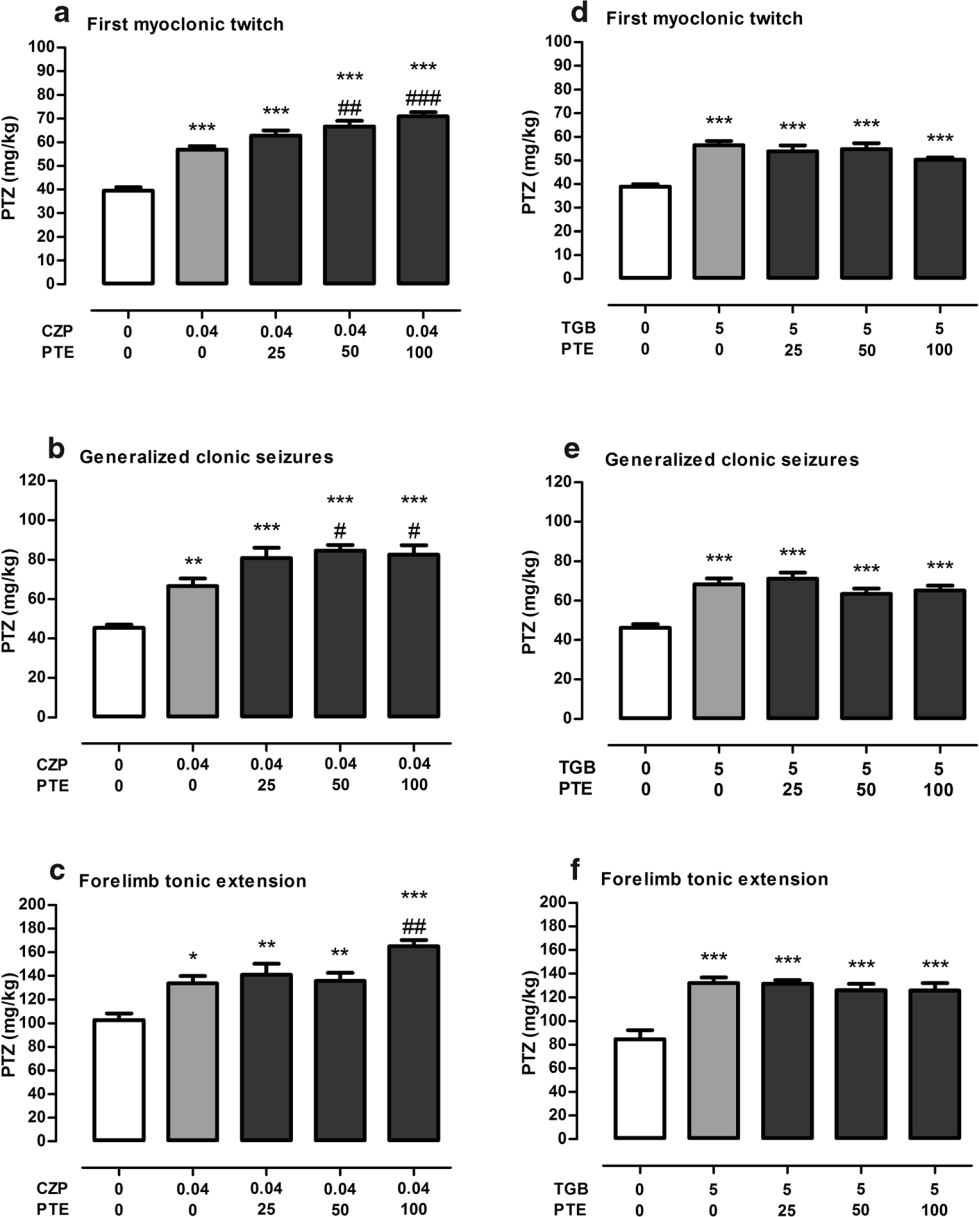
Effect of PTE on the anticonvulsant activity of CBZ (**a**–**c**) and TGB (**d**–**f**) in the *iv* PTZ seizure threshold test in mice. TGB was administered 15 min, while CZP and PTE were injected 30 min before the iv PTZ test. All the drugs were administered *ip* at a constant volume of 10 ml per kg body weight. Animals in the negative control groups received vehicle (5% Tween 80). Each experimental group consisted of 9–13 animals. Data are presented as the mean threshold dose of PTZ (in mg/kg body weight) + SEM and were analyzed using one-way ANOVA (CZP first myoclonic twitch *F*(4,54) = 42.60, *p* < 0.0001; generalized clonic seizures *F*(4,48) = 16.99, *p* < 0.0001; forelimb tonic extension *F*(4,48) = 11.02, *p* < 0.0001; TGB: first myoclonic twitch *F*(4,50) = 15.13, *p* < 0.0001; generalized clonic seizures *F*(4,52) = 13.49, *p* < 0.0001; forelimb tonic extension *F*(4,47) = 12.05, *p* < 0.0001) with the Tukey post hoc test. *p < 0.05, **p < 0.01, ***p < 0.001 vs. the negative control (5% Tween-treated) group; #*p* < 0.05, ##*p* < 0.01, ###*p* < 0.001 vs. CZP-treated group. CZP, clonazepam; PTE, pterostilbene; PTZ, pentetrazole, TGB, tiagabine

Figure 1a shows the effect of CZP (at a dose of 0.04 mg/kg) and its combinations with PTE (at doses ranging from 25 to 100 mg/kg) on threshold for the first myoclonic twitches in the *iv* PTZ test in mice (one-way ANOVA *F*(4,54) = 42.60, *p* < 0.0001). In groups of animals treated with CZP alone and its combinations with PTE, thresholds for these seizures were significantly higher than in the control (5% Tween-treated) group (*p* < 0.001). Additionally, statistical analysis revealed that anticonvulsant effect of CZP was significantly potentiated by PTE at a dose of 50 and 100 mg/kg (*p* < 0.01 and *p* < 0.001 vs. CZP-treated group, respectively).

Both CZP alone and its combinations with PTE significantly increased generalized clonic seizure thresholds in comparison with the control (5% Tween-treated) group (Fig. 1b; one-way ANOVA *F*(4,48) = 16.99, *p* < 0.0001). Statistically significant differences were also noted between groups of animals injected with a combination of CZP and PTE at doses of 50 and 100 mg/kg and a group of animals which received an injection of CZP alone (*p* < 0.05).

Effect of CZP and its combinations with PTE on the threshold for the onset of forelimb tonus in the *iv* PTZ test in mice is shown in Fig. 1c (one-way ANOVA *F*(4,48) = 11.02, *p* < 0.0001). A statistically significant increase in tonic seizure thresholds in comparison to the control (5% Tween-treated) group was noted both in the group treated with CZP alone and groups injected with a combination of this antiepileptic drug with PTE. Furthermore, PTE administered at a dose of 100 mg/kg significantly enhanced the anticonvulsant effect of CZP (*p* < 0.01) against tonic seizures.

Influence of TGB administered at a dose of 5 mg/kg as well as its combinations with PTE at doses ranging from 25 to 100 mg/kg on the seizure thresholds in the *iv* PTZ test in mice are presented in Fig. 1 d–f. Both TGB alone and its combinations with PTE increased the thresholds for the first myoclonic twitch (one-way ANOVA *F*(4,50) = 15.13, *p* < 0.0001), generalized clonic seizures with loss of righting reflex (one-way ANOVA: *F*(4,52) = 13.49, *p* < 0.0001), and tonic forelimb extension (one-way ANOVA *F*(4,47) = 12.05, *p* < 0.0001) in comparison to the control (5% Tween-treated) group. However, statistical analysis did not reveal significant differences between groups which were treated with TGB alone and groups which were injected with a combination of this drug with PTE (*p* > 0.05).

### Influence of PTE on the Anticonvulsant Activity of CBZ and TPM in the MES Test

**Fig. 2:**
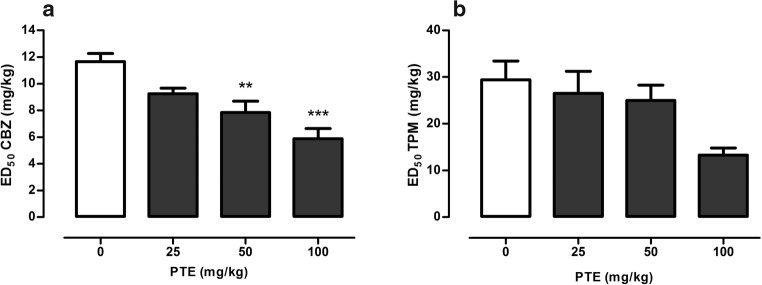
Effect of PTE on the anticonvulsant activity of CBZ (**a**) and TPM (**b**) in the MES test in mice. PTE and CBZ were administered 30 min, while TPM 60 min before the MES test. All the drugs were administered *ip* at a constant volume of 10 ml per kg body weight. Animals in the control group received antiepileptic drug (CBZ or TPM) alone, while other experimental groups received the respective antiepileptic drug in combination with PTE. Data are presented as ED doses (the doses which protect 50% of the tested animals from the maximal electroshock convulsions) + SEM and were analyzed using one-way ANOVA (CBZ *F*(3,68) = 9.70, *p* < 0.0001; TPM *F*(3,68) = 1.16, *p* = 0.333) with the Tukey post hoc test. **p < 0.01, ***p < 0.001 vs. control (CBZ-treated) group. CBZ, carbamazepine; PTE, pterostilbene, TPM, topiramate

Effect of PTE on the anticonvulsant activity of CBZ and TPM in the MES test in mice is presented in Fig. 2.

One-way ANOVA (*F*(3,68) = 9.70, *p* < 0.0001) revealed a statistically significant influence of PTE on the protective activity of CBZ in the MES test (Fig. 2a). ED_50_ value of CBZ in the control group was 11.66 ± 0.61 mg/kg, while in the group which was cotreated with PTE at a dose of 50 mg/kg was reduced to 7.83 ± 0.86 mg/kg (*p* < 0.01). The highest tested dose of PTE, i.e., 100 mg/kg, decreased ED_50_ value of CBZ by ~50% (*p* < 0.0001).

PTE at doses ranging from 25 to 100 mg/kg did not significantly influence the anticonvulsant activity of TPM in the MES test in mice (Fig. 2b, one-way ANOVA *F*(3,68) = 1.16, *p* = 0.333).

### Influence of PTE on the Anticonvulsant Activity of VPA and OXC in the 6 Hz Test

**Fig. 3:**
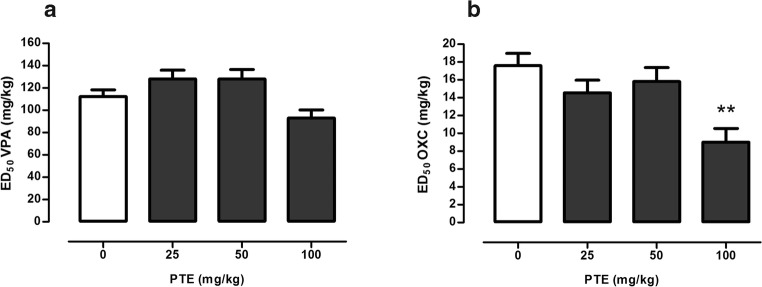
Effect of PTE on the anticonvulsant activity of VPA (**a**) and OXC (**b**) in the 6 Hz psychomotor seizure test in mice. VPA was administered 15 min, while PTE and OXC 30 min before the 6 Hz test. All the drugs were administered *ip* at a constant volume of 10 ml per kg body weight. Animals in the control group received antiepileptic drug (VPA or OXC) alone, while other experimental groups received the respective antiepileptic drug in combination with PTE. Data are presented as ED doses (the doses which protect 50% of the tested animals from the psychomotor seizures) + SEM and were analyzed using one-way ANOVA (VPA *F*(3,69) = 4.43, *p* = 0.007; OXC *F*(3,68) = 5.31, *p* = 0.002) with the Tukey post hoc test. **p < 0.01 vs. control (OXC-treated) group. OXC, oxcarbazepine; PTE, pterostilbene; VPA, valproate

Effect of PTE (at doses of 25–100 mg/kg) on the anticonvulsant activity of VPA in the 6 Hz test is depicted in Fig. 3a. Although one-way ANOVA revealed statistically significant changes between experimental groups (one-way ANOVA *F*(3,69) = 4.43, *p* = 0.007), there were no statistically significant differences between control (VPA-treated) group and groups of animals which were injected with combinations of this antiepileptic drug with PTE.

PTE significantly enhanced the protective properties of OXC in the 6 Hz test in mice (Fig. 3b; one-way ANOVA *F*(3,68) = 5.31, *p* = 0.002). ED_50_ value of OXC in the control group was 17.61 ± 1.36 mg/kg, while in the group that was co-administered with OXC and PTE at a dose of 100 mg/kg was reduced to 9.02 ± 1.54 mg/kg (~49%).

### Effect of the Studied Antiepileptic Drugs and their Combinations with PTE in the Chimney Test and Grip-Strength Test

Influence of the studied antiepileptic drugs and their combinations with PTE on the skeletal muscles strength and motor coordination in mice is presented in Table 1.

**Table 1:** Influence of the studied antiepileptic drugs and their combinations with PTE on the muscular strength and motor coordination in mice

Treatment (mg/kg)	Neuromuscular strength (mN/g)	Impairment of motor coordination (%)	*N*
A			
Control (5% Tween)	28.80 ± 1.1	0	12
CZP (0.04)	28.31 ± 1.6	0	12
CZP (0.04) + PTE (25)	26.25 ± 1.1	0	11
CZP (0.04) + PTE (50)	27.71 ± 0.9	0	12
CZP (0.04) + PTE (100)	27.99 ± 1.2	0	12
	One-way ANOVA *F*(4,54) = 0.604, *p* = 0.661		
B			
Control (5% Tween)	29.09 ± 1.8	0	12
TGB (5)	29.18 ± 0.9	0	12
TGB (5) + PTE (25)	29.37 ± 0.9	9	11
TGB (5) + PTE (50)	28.66 ± 1.1	8	12
TGB (5) + PTE (100)	28.23 ± 0.9	0	12
	One-way ANOVA *F*(4,55) = 0.16, *p* = 0.958		
C			
Control (5% Tween)	30.60 ± 0.9	0	10
CBZ (5.9)	30.38 ± 1.1	0	10
CBZ (5.9) + PTE (100)	26.72 ± 1.1*#	10	10
	One-way ANOVA *F*(2,27) = 4.56, *p* = 0.020		
D			
Control (5% Tween 80)	32.49 ± 0.9	0	10
TPM (29.4)	30.45 ± 1.3	0	10
TPM (29.4) + PTE (100)	31.20 ± 1.0	0	10
	One-way ANOVA *F*(2,27) = 0.93, *p* = 0.409		
E			
Control (5% Tween)	28.53 ± 1.2	0	10
VPA (93)	28.09 ± 1.7	0	10
VPA (93) + PTE (100)	26.20 ± 1.1	0	10
	One-way ANOVA *F*(2,27) = 0.82, *p* = 0.451		
F			
Control (5% Tween)	30.60 ± 0.9	0	10
OXC (9.02)	31.77 ± 1.5	0	10
OXC (9.02) + PTE (100)	30.48 ± 1.0	0	10
	One-way ANOVA *F*(2,27) = 0.39, *p* = 0.683		

VPA and TGB were administered 15 min before the tests, PTE, CBZ, OXC, and CZP, 30 min, while TPM, 60 min. Control group received the vehicle. All solutions/suspensions were injected *ip*. Each experimental group consisted of 10–12 animals. Results from the grip strength test are presented as the mean ± SEM grip strengths in millinewtons per gram of mouse body weight (mN/g) and were analyzed with one-way ANOVA followed by the Tukey post hoc test. Data from the chimney test are presented as a percentage of animals with impairment of motor coordination and were compared using the Fisher exact probability test. *p < 0.05 vs. control (5% Tween-treated) group; #*p* < 0.05 vs. CBZ-treated group. *CBZ* carbamazepine, *CZP* clonazepam, *OXC* oxcarbazepine, *PTE* pterostilbene, *TGB* tiagabine, *VPA* valproate

A statistically significant decrease in muscles strength was noted in the group of animals treated with a combination of CBZ (5.9 mg/kg) and PTE (100 mg/kg) (Tukey’s test *p* < 0.05 both vs. 5% Tween-treated and CBZ-treated groups, Table 1 part C). The remaining antiepileptic drugs and their combinations with PTE did not statistically affect the strength of muscles in mice.

In addition, antiepileptic drugs at the studied doses and their combinations with PTE did not significantly influence motor coordination in the chimney test in mice (*p* > 0.05).

### Effect of PTE on the Serum and Total Brain Concentrations of CZP, CBZ, and OXC

Effect of PTE on the serum and total brain concentrations of CZP, CBZ, and OXC (antiepileptic drugs which activity was changed by PTE in seizure tests) is presented in Table 2.

**Table 2:** Effect of PTE on serum and total brain concentration of CZP, CBZ and OXC

Treatment (mg/kg)	Serum concentration	Total brain concentration
A		
CZP (0.04)	16.40 ± 2.12 (ng/ml)	4.45 ± 0.61 (ng/g tissue)
CZP (0.04) + PTE (100)	14.28 ± 1.20 (ng/ml)	4.40 ± 0.32 (ng/g tissue)
	Student’s *t* test: *p* = 0.1977	Student’s *t* test: *p* = 0.4705
B		
CBZ (5.9)	2.06 ± 0.10 (μg/ml)	3.72 ± 0.45 (μg/g tissue)
CBZ (5.9) + PTE (100)	2.41 ± 0.06 (μg/ml)**	5.45 ± 0.36 (μg/g tissue)**
	Student’s *t* test: *p* = 0.0043	Student’s *t* test: *p* = 0.0039
C		
OXC (9.02)	2.97 ± 0.19 (μg/ml)	3.46 ± 0.27 (μg/g tissue)
OXC (9.02) + PTE (100)	4.23 ± 0.46 (μg/ml)*	5.47 ± 0.58 (μg/g tissue)**
	Student’s *t* test: *p* = 0.0104	Student’s *t* test: *p* = 0.0027

PTE, CZP, CBZ, and OXC were administered *ip* 30 min before the samples collection. Each experimental group consisted of 10 mice. Results are presented as the mean ± SEM. Serum and brain concentrations of the studied antiepileptic drugs were analyzed using the Student *t* test. *p < 0.05, **p < 0.01 vs. control (treated with antiepileptic drug alone) group. *CBZ* carbamazepine, *CZP* clonazepam, *OXC* oxcarbazepine, *PTE* pterostilbene

PTE administered at a dose of 100 mg/kg changed neither serum nor total brain concentration of CZP (Table 2 part A). Co-administration of PTE at a dose of 100 mg/kg with CBZ at a dose of 5.9 mg/kg significantly increased both serum and total brain concentrations of CBZ (Table 2 part B). Similarly, PTE at a dose of 100 mg/kg significantly raised the level of OXC in serum as well as in the brain (Table 2 part C).

## Discussion

PTE has recently attracted much attention due to a wide variety of biological and pharmacological properties determined both in preclinical and clinical studies. Among numerous beneficial effects, its influence on the central nervous system related processes was also noted. PTE improves memory and cognition processes (Hou et al. 2014; Naik et al. 2017) and showed anxiolytic activity (Al Rahim et al. 2013) in animal models. Additionally, we showed the anticonvulsant activity of PTE in the zebrafish PTZ assay and three acute seizure threshold tests in mice, i.e., in the *iv* PTZ, MEST, and 6 Hz psychomotor seizure tests (Nieoczym et al. 2019). In our present study, we investigated the influence of PTE on the activity of some antiepileptic drugs in these seizure tests. Although results of murine studies could not be directly transferred to the clinical practice, our results could serve as an indication to assess the safety of using PTE by patients treated with antiepileptic drugs and whether it could be used as add-on therapy.

In our study, we used three different seizure tests which allowed us to analyze the influence of PTE on the action of some antiepileptic drugs in relation to different kinds of seizures. The *iv* PTZ test is considered a model of generalized absence seizures (Mandhane et al. 2007), the MES test corresponds to the generalized tonic-clonic convulsions (Castel-Branco et al. 2009), while the 6 Hz test was described as a model of psychomotor seizures (Barton et al. 2001) in humans. All of these tests are widely used in the preclinical study of compounds with potential antiepileptic properties. Our results demonstrated that PTE significantly enhanced the anticonvulsant action of CZP in the *iv* PTZ test, CBZ in the MES test, and OXC in the 6 Hz-induced psychomotor seizure model in mice. There was no statistically significant influence of PTE on the anticonvulsant properties of TGB, TPM, and VPA in the respective experimental models.

Using combinations of some drugs or drugs and diet supplements, which are often taken without medical recommendation and may not be reported to physicians, gives an opportunity to produce pharmacological interactions with both beneficial and harmful effects in clinical response. Unwanted effects might include a reduction in therapeutic effects of the drug as well as worsen and/or induction of some side effects which might lead to treatment discontinuation. Pharmacological interactions of drugs may have pharmacokinetic or pharmacodynamic character. Pharmacodynamic interactions concern changes in drug activity in the area of target tissue, i.e., influence of agonist and antagonist at the same receptor in the brain, while pharmacokinetic interactions are noted when one drug affects the concentration of another drug by influence on its absorption, distribution, and/or elimination (Corrie and Hardman 2014; Zaccara and Perucca 2014). To determine the kinds of pharmacological interactions occurred between PTE and CZP, CBZ and OXC, we evaluated serum and total brain concentration of these antiepileptic drugs. Our study revealed that the enhancement of the anticonvulsant activity of CBZ and OXC resulted from the increase in both serum and total brain concentrations of these drugs, which suggests that noted interactions were pharmacokinetic in nature, although the pharmacodynamic interactions might not be completely excluded. Contrary to CBZ and OXC, PTE did not change serum and total brain concentration of CZP, which indicates that interaction between these two drugs might have pharmacodynamic character. This kind of interaction might be desirable in clinical practice because it enables to enhance therapeutic activity of drug without a need for using its higher doses.

We could hypothesize that pharmacokinetic interactions between PTE and other drugs, including antiepileptic drugs, i.e., CBZ and OXC, might also occur in the clinical practice. It was noted that PTE is able to affect the activity of cytochrome P450 enzyme system which plays an important role in phase I metabolism of many drugs and xenobiotics and catalyzes mainly their oxidative metabolism, i.e., it inhibited CYP1A1, CYP1A2, CYP2C8, and CYP3A4 (Albassam and Frye 2019; Hyrsova et al. 2019; Mikstacka et al. 2007). Hyrsova et al. (2019) reported that PTE inhibited CYP3A4 activity with the inhibitory constant (K_i_) lower than 10 μM, while Albassam and Frye (2019) detected that concentration of PTE that results in 50% inhibition (IC_50_) of CYP2C8 activity is less than 20 μM. Although the content of PTE in the diet might be too low to affect cytochrome P450 activity, its supplementation might lead to a sufficient increase in its concentration in the body and impact on the activity of these enzymes, which would lead to pharmacological interactions with drugs (Albassam and Frye 2019; Hyrsova et al. 2019). Furthermore, our previous study (Nieoczym et al. 2019) revealed that serum and brain concentration of PTE after its administration at a dose of 50 mg/kg is enough for the inhibition of cytochrome P450 isoenzymes in mice (detailed data available in Nieoczym et al. 2019).

In humans, CBZ is metabolized to carbamazepine-10,11-epoxide, and this reaction is catalyzed primarily by CYP3A4, CYP2C8, and CYP3A5 isoenzymes (Kerr et al. 1994; Pearce et al. 2008). Since PTE might influence the activity of both CYP3A4 and CYP2C8 isoenzymes, pharmacokinetic interactions between this compound and CBZ is also possible in clinical practice.

Although OXC is structurally similar to CBZ, its metabolism is different because it is rapidly reduced by cytosol aryl ketone reductase to its 10-monohydroxy derivative—MHD, which is next cleared by glucuronide conjugation. Glucuronidation, linkage of glucuronic acid component of uridine diphosphate glucuronic acid (UDP) to substrate, is catalyzed by a family of uridine 5′-diphospho-glucuronosyl transferases (UDP-glucuronosyltransferases, UGTs) (May et al. 2003; Rowland et al. 2013). It has been recently reported that PTE significantly inhibited UGT1A6 activity with IC_50_ value of approximately 15 μM (Albassam and Frye 2019). As in the case of cytochrome P450 isoenzymes, supplementation of PTE in humans may lead to inhibition of UGTs activity and pharmacokinetic interactions (Albassam and Frye 2019).

Mechanism of antiepileptic drugs action is often related to their influence on the different membrane ion channels, which consequently affects neurons excitability. Among the antiepileptic drugs which were investigated in our study, CBZ, OXC, and TPM work mainly by inhibiting sodium and calcium channels (Lasoń et al. 2011). There are no studies concerning influence of PTE on membrane channels activity, but studies of RES revealed that it might inhibit some sodium and calcium channels in neurons (Kim et al. 2005; Li et al. 2005). RES, the structure of which is quite similar to PTE, was previously demonstrated to activate the activity of large-conductance calcium-activated potassium channels in different types of cells including central neurons (Li et al. 2000; Wang et al. 2016). Whether PTE exerts anticonvulsant effects on potassium channels remains to be further explored. The above data suggest that the interactions between PTE and the studied antiepileptic drugs might be also related to the interaction with the same ion channels in the neuronal cell membranes, and therefore, pharmacodynamic interactions of the studied stilbene with CBZ and OXC are also possible. Detailed studies on the effects of PTE on the respective ion channels activity are required.

Pharmacokinetic interactions are rather not desired in clinical practice because drug accumulation in body tissues might not only increase its therapeutic effect but, above all, might potentiate its toxicity and lead to the appearance and/or enhancement of side effects. We noted attenuation of muscular strength in mice administered with a combination of PTE and CBZ, but other side effects are also possible. Furthermore, we did not detect changes in the muscular strength and motor coordination in mice co-treated with OXC and PTE; however, we could not exclude the occurrence of other side effects, i.e., related to memory and/or concentration.

In conclusion, our data suggest that PTE might affect the activity of some antiepileptic drugs. It enhanced the anticonvulsant effect of CZP as a result of pharmacodynamic interactions, but in the case of CBZ and OXC, the increase in anticonvulsant effect resulted from pharmacokinetic interactions. However, the presence of pharmacokinetic interactions does not exclude completely the presence of pharmacodynamic interactions. Although our study was carried out in mice and the results could not be directly transferred to humans, caution should be taken in using PTE in combination with CBZ and OXC. On the other hand, combination of PTE with CZP might have beneficial therapeutic effects. Further studies are required to explore the influence of PTE on the activity of antiepileptic drugs and to evaluate both beneficial and harmful effects of using PTE in patients with epilepsy.

## Abbreviations

CBZ: Carbamazepine
CZP: Clonazepam
ED_50_: Dose which protects 50% of the tested animals from the seizures
*ip*: Intraperitoneally
*iv*: Intravenously
MES: Maximal electroshock
OXC: Oxcarbazepine
PTE: Pterostilbene
PTZ: Pentetrazole
*sc*: Subcutaneously
SEM: Standard error of the mean
TGB: Tiagabine
TPM: Topiramate
VPA: Valproate
